# A body shape index is associated with endothelial dysfunction in both men and women

**DOI:** 10.1038/s41598-021-97325-0

**Published:** 2021-09-09

**Authors:** Masato Kajikawa, Tatsuya Maruhashi, Shinji Kishimoto, Takayuki Yamaji, Takahiro Harada, Yu Hashimoto, Yiming Han, Aya Mizobuchi, Gaku Aoki, Kenichi Yoshimura, Kazuaki Chayama, Chikara Goto, Farina Mohamad Yusoff, Ayumu Nakashima, Yukihito Higashi

**Affiliations:** 1 Division of Regeneration and Medicine, Medical Center for Translational and Clinical Research, Hiroshima University Hospital, Hiroshima, Japan; 2 Department of Cardiovascular Regeneration and Medicine, Research Institute for Radiation Biology and Medicine, Hiroshima University, 1-2-3 Kasumi, Minami-ku, Hiroshima, 734-8551 Japan; 3 Department of Cardiovascular Medicine, Hiroshima University Graduate School of Biomedical Sciences, Hiroshima, Japan; 4 Department of Biostatistics, Medical Center for Translational and Clinical Research, Hiroshima University Hospital, Hiroshima, Japan; 5 Department of Gastroenterology and Metabolism, Institute of Biomedical and Health Sciences, Graduate School of Biomedical and Health Sciences, Hiroshima University, Hiroshima, Japan; 6 Department of Physical Therapy, Hiroshima International University, Hiroshima, Japan; 7 Department of Stem Cell Biology and Medicine, Hiroshima University Graduate School of Biomedical Sciences, Hiroshima, Japan

**Keywords:** Biomarkers, Cardiology, Risk factors

## Abstract

A body shape index (ABSI) was proposed for estimation of abdominal adiposity. ABSI has been reported to have associations with cardiovascular risk factors and cardiovascular events. However, there is no information on the association between ABSI and endothelial function. We examined cross-sectional associations of ABSI with endothelial function in 8823 subjects (6773 men and 2050 women). Subjects with a lower quartile of flow-mediated vasodilation (FMD) were defined as subjects having endothelial dysfunction. Pearson’s correlation coefficient analysis revealed that ABSI was negatively correlated with FMD (men, r = − 0.23, P = 0.003; women, r = − 0.32, P < 0.001). The areas under the curves of ABSI and body mass index to predict endothelial dysfunction were 0.64 (95% confidence interval [CI] 0.62–0.65) and 0.58 (95% CI 0.57–0.60) in men, and 0.68 (95% CI 0.66–0.71) and 0.59 (95% CI 0.56–0.61) in women, respectively. The cutoff values of ABSI for predicting subjects with endothelial dysfunction were 0.0796 (sensitivity, 55.2%; specificity, 65.5%) in men and 0.0823 (sensitivity, 56.2%; specificity, 73.4%) in women. Multivariate analysis revealed that an ABSI value higher than the cutoff value remained an independent predictor of endothelial dysfunction in both sexes. The results of our study suggest that ABSI calculation should be performed for evaluation of risk of cardiovascular events in both men and women.

**Clinical trial registration information** URL for Clinical Trial: https://www.umin.ac.jp/ctr/index.htm; Registration Number for Clinical Trial: UMIN000012952 (01/05/2010).

## Introduction

Endothelial dysfunction is the initial stage of atherosclerosis and is recognized as a key player in the development of atherosclerosis, resulting in cardiovascular complications^1,2^. Measurement of flow-mediated vasodilation (FMD) of the brachial artery as a marker of endothelial function has been widely used in clinical practice to evaluate endothelial function^3–6^. Several investigators have reported associations between endothelial dysfunction and cardiovascular events^7–10^.

Body mass index (BMI) is a simple screening tool for classifying obesity. Although an increase in BMI is known to be associated with the prevalence of cardiovascular risk factors, the effect of an increase in BMI on the incidence of cardiovascular events is still controversial^6,11–13^. Numerous studies have suggested that higher BMI is associated with lower risk of mortality in patients with coronary artery disease, an association that is known as the “obesity paradox”^14^. One possible explanation for the obesity paradox is that BMI does not reflect regional body fat distribution and cannot distinguish muscle and fat mass. Indeed, several investigators have suggested that abdominal obesity and abdominal deposition of fat are more strongly associated with cardiovascular risk factors than is BMI^15,16^. It is expected that evaluation of abdominal obesity would improve the predictive value of future cardiovascular events compared with BMI. However, there are no accepted simple criteria for evaluation of abdominal obesity.

Waist circumference is one of the simple methods evaluating abdominal obesity. Since waist circumference is sensitive to body size (height, weight, and BMI), measurement of waist circumference cannot provide accurate information on the abdominal deposition of fat^17^. Krakauer et al. proposed a body shape index (ABSI) for estimation of abdominal adiposity^18^. ABSI has been shown to be associated with cardiovascular risk factors, mortality, and cardiovascular events in several ethnic groups^18–23^. However, there is no information on the association between ABSI and endothelial function. In this study, we evaluated the association between ABSI and endothelial function determined by measurement of FMD in a general population.

## Results

### Baseline characteristics

Baseline characteristics of all subjects are summarized in Table 1. Of the 8823 subjects, 6773 (77%) were men, 4177 (47%) had hypertension, 4664 (53%) had dyslipidemia, 1107 (13%) had diabetes mellitus, 881 (10%) had previous cardiovascular disease, and 2482 (28%) were current smokers. The mean value of ABSI was 0.0792 ± 0.0048 (men, 0.0788 ± 0.0041; women, 0.0803 ± 0.0064). There were significant differences between men and women in age, BMI, height, weight, waist circumference, systolic blood pressure, diastolic blood pressure, heart rate, total cholesterol, triglycerides, high-density lipoprotein cholesterol, glucose, prevalence of hypertension and diabetes mellitus, smoking history, history of cardiovascular disease, use of anti-hypertensive drugs, lipid-lowering drugs, and anti-diabetic drugs, Framingham risk score, and ABSI.

**Table 1:** Clinical characteristics of the subjects.

Variables	Total (n = 8823)	Men (n = 6773)	Women (n = 2050)	P value
Age (year)	52 ± 13	51 ± 13	55 ± 15	< 0.001
Body mass index (kg/m^2^)	23.5 ± 3.4	23.8 ± 3.3	22.7 ± 3.8	< 0.001
Height (m)	1.66 ± 0.09	1.69 ± 0.06	1.55 ± 0.07	< 0.001
Weight (kg)	65.2 ± 11.9	68.3 ± 10.6	54.7 ± 9.6	< 0.001
Waist circumference (cm)	83.6 ± 9.5	84.7 ± 8.9	79.9 ± 10.7	< 0.001
Systolic blood pressure (mmHg)	128 ± 17	129 ± 16	124 ± 19	< 0.001
Diastolic blood pressure (mmHg)	79 ± 12	80 ± 12	75 ± 12	< 0.001
Heart rate (bpm)	65 ± 11	64 ± 11	67 ± 11	< 0.001
Total cholesterol (mmol/L)	5.17 ± 0.88	5.15 ± 0.88	5.22 ± 0.91	0.008
Triglycerides (mmol/L)	1.46 ± 1.07	1.56 ± 1.15	1.15 ± 0.68	< 0.001
HDL-C (mmol/L)	1.53 ± 0.41	1.47 ± 0.39	1.71 ± 0.41	< 0.001
LDL-C (mmol/L)	3.03 ± 0.78	3.03 ± 0.78	3.03 ± 0.41	0.94
Glucose (mmol/L)	5.66 ± 1.33	5.72 ± 1.33	5.55 ± 1.39	< 0.001
**Medical history, n (%)**				
Hypertension	4177 (47)	3132 (46)	1045 (51)	< 0.001
Dyslipidemia	4664 (53)	3615 (53)	1049 (51)	0.08
Diabetes mellitus	1107 (13)	786 (12)	321 (16)	< 0.001
Previous cardiovascular disease	881 (10)	712 (11)	169 (8)	0.002
Current smoker	2482 (28)	2321 (34)	161 (8)	< 0.001
**Medications, n (%)**				
Anti-hypertensive therapy	2869 (33)	2025 (30)	844 (41)	< 0.001
Any lipid modification therapy	1558 (18)	1049 (15)	509 (25)	< 0.001
Anti-hyperglycemic therapy	781 (9)	557 (8)	224 (11)	< 0.001
Framingham risk score (%)	8.6 ± 7.6	9.3 ± 8.0	6.1 ± 5.7	< 0.001
FMD (%)	5.7 ± 3.2	5.8 ± 3.1	5.7 ± 3.7	0.92
A body shape index	0.0792 ± 0.0048	0.0788 ± 0.0041	0.0803 ± 0.0064	< 0.001

Results are presented as mean ± SD for continuous variables and percentages for categorical variables.

*HDL-C* high-density lipoprotein cholesterol, *LDL-C* low-density lipoprotein cholesterol, *FMD* flow-mediated vasodilation.

### Relationships between ABSI and cardiovascular risk factors

**Figure 1:**
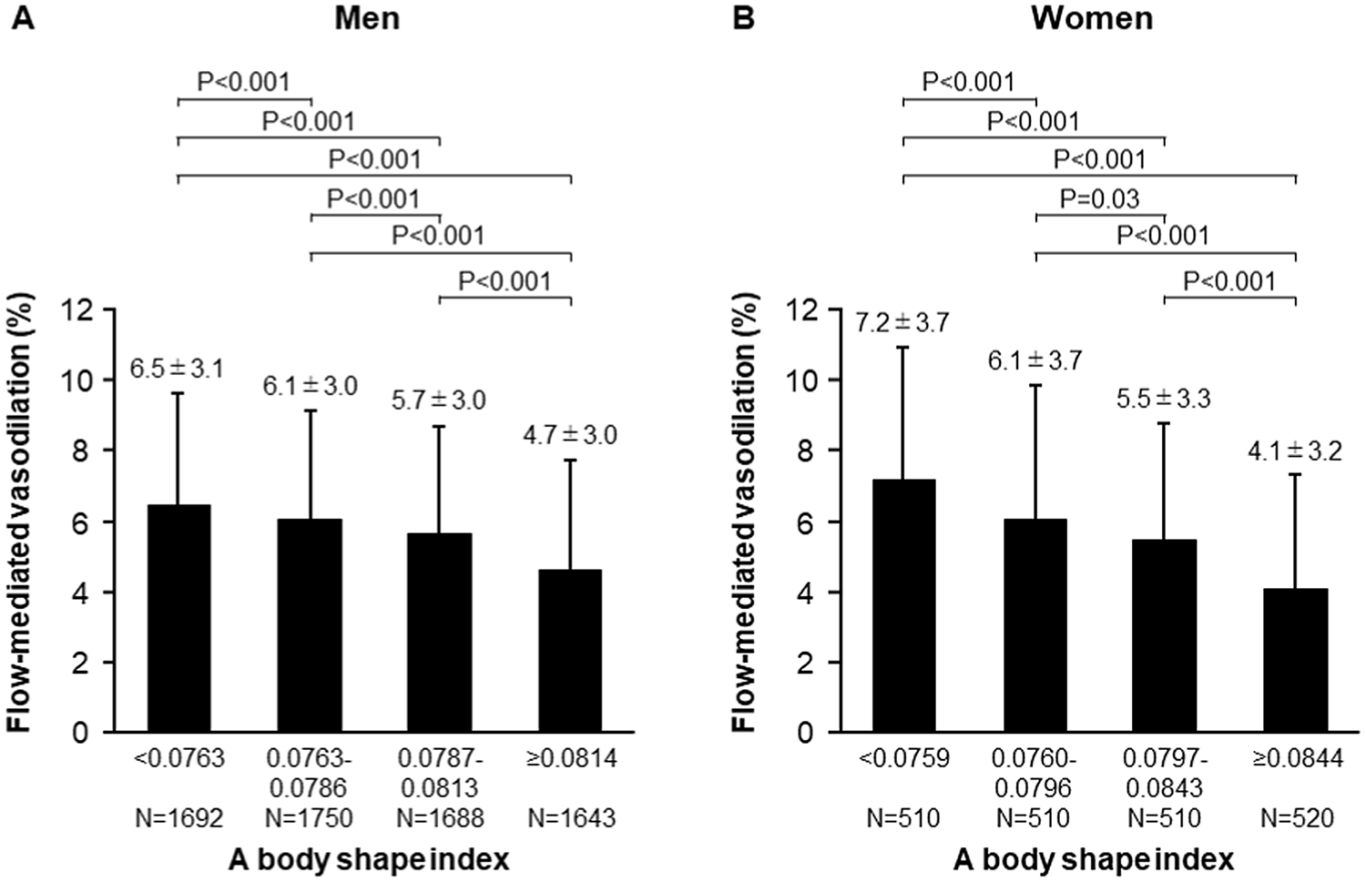
Bar graphs showing flow-mediated vasodilation according to a body shape index in men (**A**) and in women (**B**).

**Figure 2:**
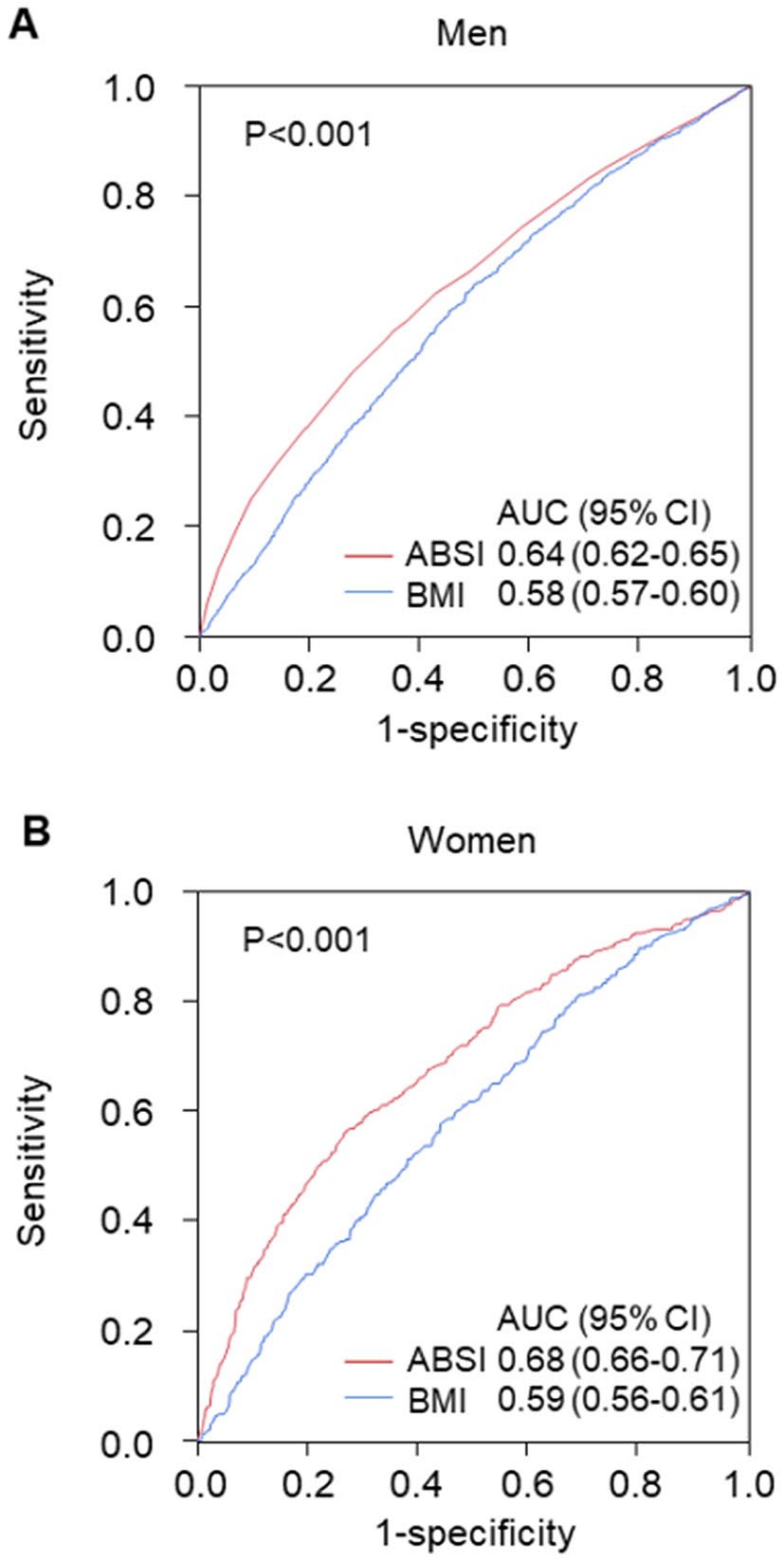
Receiver-operator characteristic curves of a body shape index (ABSI) and body mass index (BMI) for patients with a low quartile of flow-mediated vasodilation (FMD) in men (**A**) and in women (**B**). Low quartile of FMD indicates less than 3.6% in men and less than 3.1% in women. P values indicate the differences in areas under the curves between ABSI and BMI to predict a low quartile of FMD. *ABSI* a body shape index, *BMI* body mass index, *AUC* area under the curve, *CI* confidence interval.

**Figure 3:**
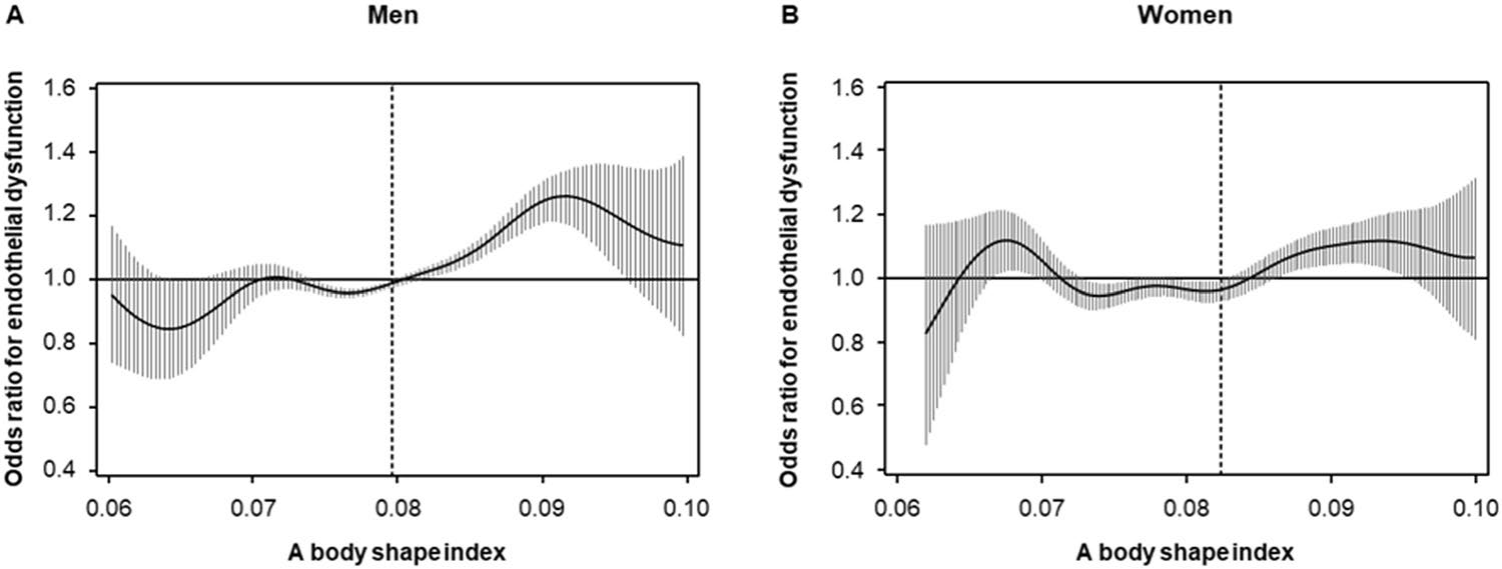
Adjusted cubic spline of the relationship between a body shape index (ABSI) and odds ratio for endothelial dysfunction in men (**A**) and in women (**B**). Endothelial dysfunction was defined as FMD of less than 3.6% in men and FMD of less than 3.1% in women. The adjusted model includes age, body mass index, and smokers. Vertical lines show 95% confidence intervals. Dashed vertical lines represent the cutoff values of ABSI for endothelial dysfunction that were derived from ROC curve analysis (0.0796 for men and 0.0823 for women).

**Figure 4:**
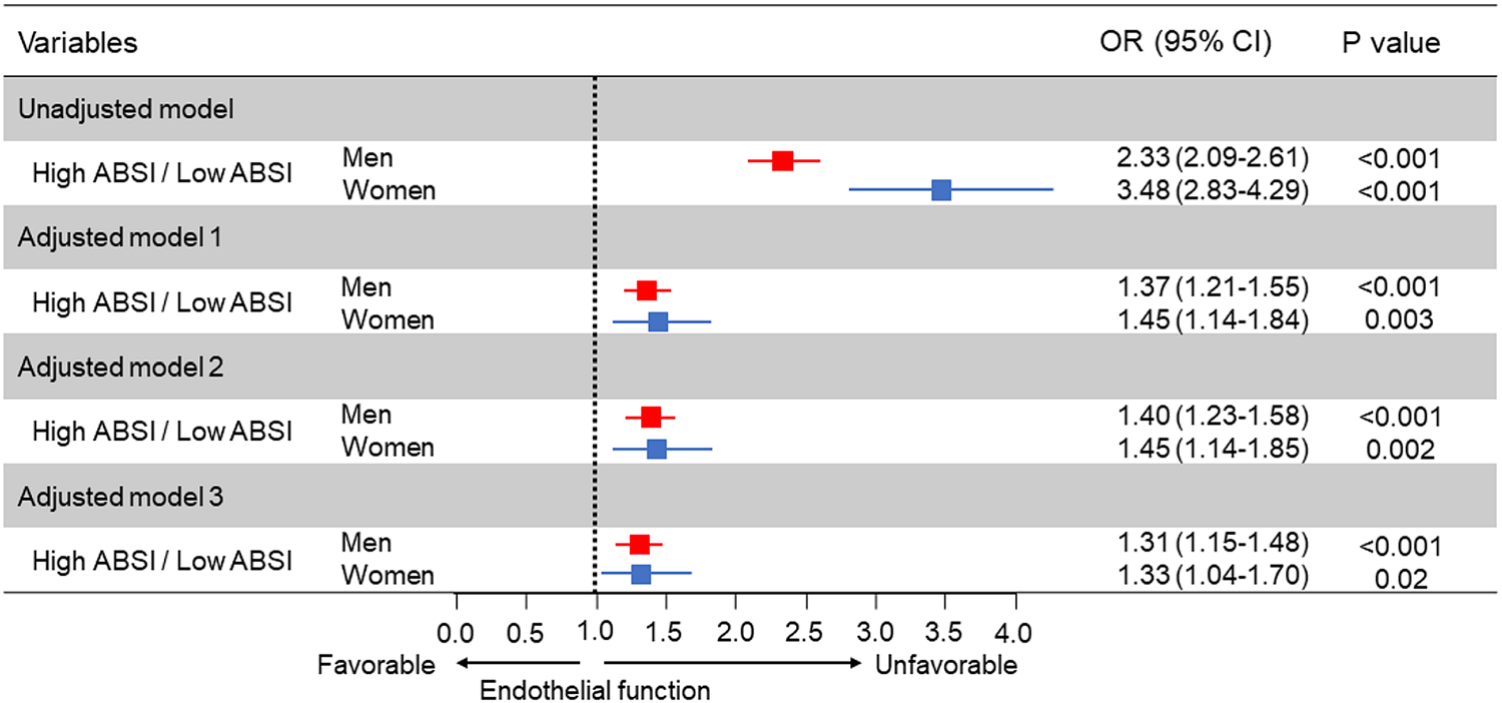
Odds ratios and 95% confidence intervals for a low quartile of flow-mediated vasodilation (FMD) of high a body shape index (ABSI) using the low ABSI group as the reference. Low quartile of FMD indicates less than 3.6% in men and less than 3.1% in women. Low ABSI indicates less than 0.0796 in men and less than 0.0823 in women. Model 1: Adjusted for age. Model 2: Adjusted for age, body mass index, and smokers. Model 3: Adjusted for age, body mass index, presence of hypertension, dyslipidemia, and diabetes, and smokers.

The associations between ABSI and variables by using Pearson’s correlation coefficients analysis are shown in Table 2. There were significant relationships between FMD and ABSI (Table 2). The correlation of ABSI and BMI for men was negligible (− 0.001) with a modest correlation in females of 0.042 (Table 2). Subjects were categorized into four quartile groups based on ABSI. Baseline characteristics of the subjects are summarized in online Supplementary Tables S1 and S2. FMD was significantly impaired in higher ABSI groups (Fig. 1). ROC curve analysis revealed that ABSI and BMI predict the low quartile of FMD with AUC values of 0.64 (95% confidence interval [CI] 0.62–0.65) and 0.58 (95% CI 0.57–0.60) in men, and 0.68 (95% CI 0.66–0.71) and 0.59 (95% CI 0.56–0.61) in women, respectively (Fig. 2). The cutoff values of ABSI and BMI for predicting subjects with endothelial dysfunction were 0.0796 (sensitivity, 55.2%; specificity, 65.5%) and 23.2 (sensitivity, 64.1%; specificity, 50.1%), respectively, in men and 0.0823 (sensitivity, 56.2%; specificity, 73.4%) and 22.4 (sensitivity, 57.9%; specificity, 55.8%), respectively, in women. The number of subjects who had ABSI values higher than the cutoff values were 2674 (39%) in men and 703 (34%) in women. An adjusted cubic spline curve described the relationship between ABSI and odds ratio for endothelial dysfunction (Fig. 3). Figure 4 and online Supplementary Fig. S1 show the crude and multivariate-adjusted odds ratios of the low quartile of FMD according to ABSI and BMI. After adjustment of various confounders, ABSI value higher than the cutoff value remained as an independent predictor of endothelial dysfunction regardless of gender, and BMI higher than the cutoff value remained as an independent predictor of endothelial dysfunction in men.

**Table 2:** Pearson’s correlation coefficients analysis of relationships between a body shape index and variables.

Variables	Men	Women
Age (year)	0.442^†^	0.549^†^
Body mass index (kg/m^2^)	− 0.001	0.042
Height (m)	− 0.121^†^	− 0.226^†^
Weight (kg)	− 0.059^†^	− 0.063*
Waist circumference (cm)	0.477^†^	0.591^†^
Systolic blood pressure (mmHg)	0.100^†^	0.220^†^
Diastolic blood pressure (mmHg)	0.040*	0.074*
Heart rate (bpm)	0.126^†^	0.112^†^
Total cholesterol (mmol/L)	− 0.043*	0.045
Triglycerides (mmol/L)	0.084^†^	0.187^†^
HDL cholesterol (mmol/L)	− 0.087^†^	− 0.130^†^
LDL cholesterol (mmol/L)	− 0.036*	0.053
Glucose (mmol/L)	0.186^†^	0.189^†^
Framingham risk score (%)	0.238^†^	0.357^†^
FMD (%)	− 0.234^†^	− 0.319^†^

*HDL* high-density lipoprotein, *LDL* low-density lipoprotein, *FMD* flow-mediated vasodilation.

Pearson’s correlation coefficients analysis of the relations between a body shape index and variables.

*P < 0.001.

^†^P < 0.0001.

### Relationships between ABSI and history of cardiovascular disease

**Figure 5:**
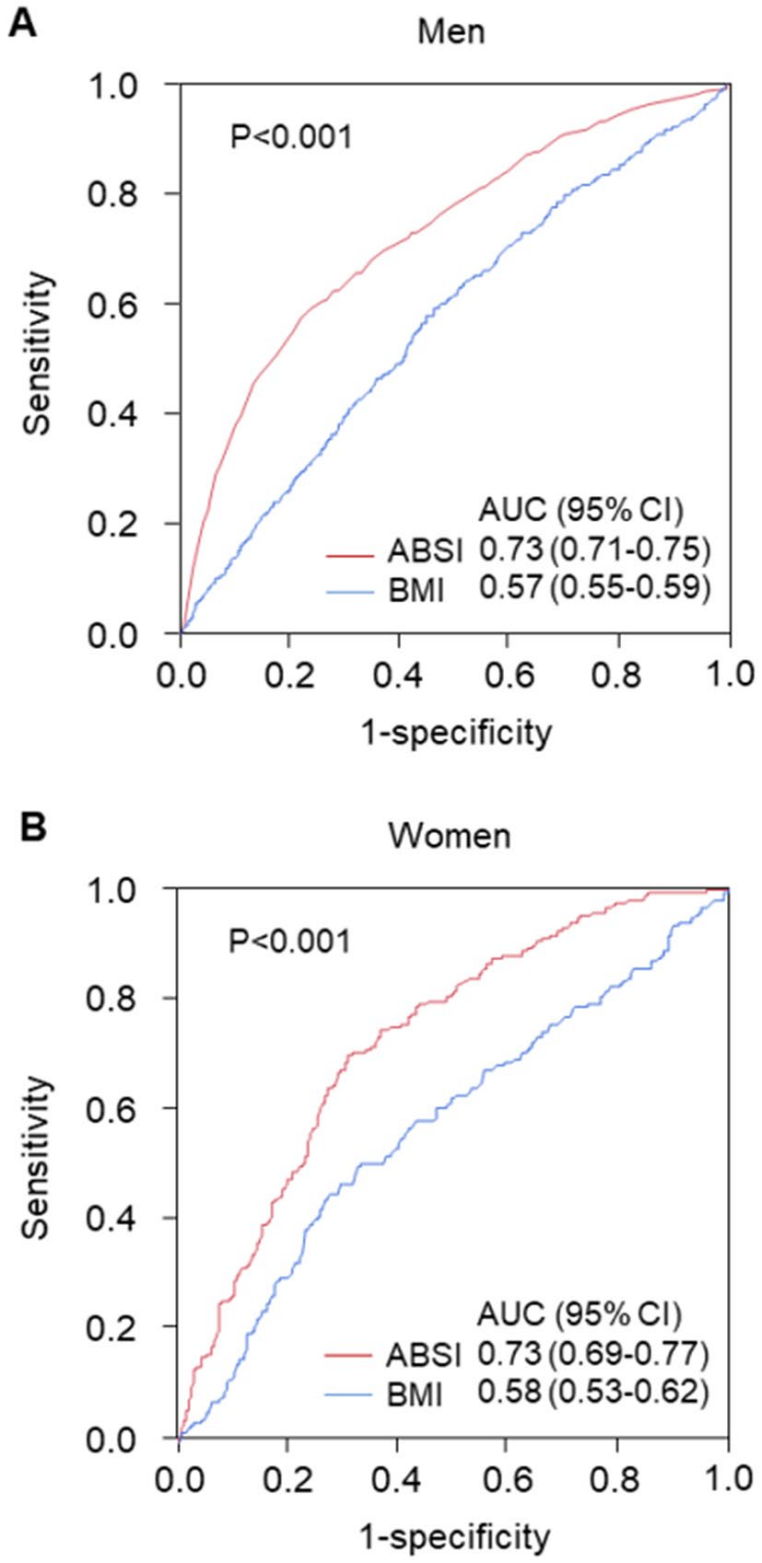
Receiver-operator characteristic curves of a body shape index (ABSI) and body mass index (BMI) for predicting patients with a history of cardiovascular disease in men (**A**) and in women (**B**). P values indicate the differences in areas under the curves between ABSI and BMI to predict patients with cardiovascular disease. *ABSI* a body shape index, *BMI* body mass index, *AUC* area under curve, *CI* confidence interval.

**Figure 6:**
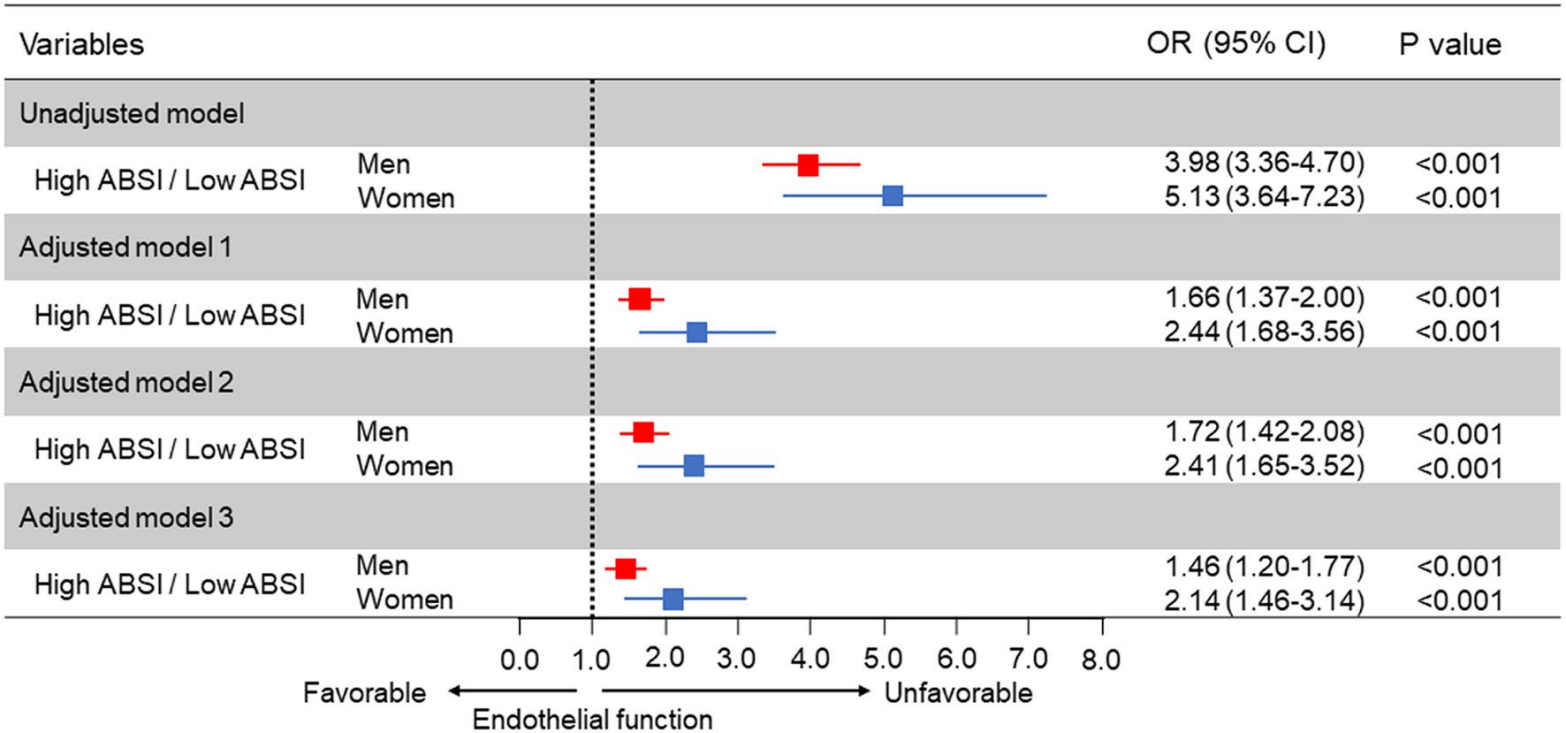
Odds ratios and 95% confidence intervals for predicting patients with a history of cardiovascular disease of high a body shape index (ABSI) using the low ABSI group as the reference. Low ABSI indicates less than 0.0796 in men and less than 0.0823 in women. Model 1: Adjusted for age. Model 2: Adjusted for age, body mass index, and smokers. Model 3: Adjusted for age, body mass index, presence of hypertension, dyslipidemia, and diabetes, and smokers.

ROC curve analysis revealed that ABSI and BMI detect a history of cardiovascular disease with AUC values of 0.73 (95% CI 0.71–0.75) and 0.57 (95% CI 0.55–0.59), respectively, in men, and 0.73 (95% CI 0.69–0.77) and 0.58 (95% CI 0.53–0.62), respectively, in women (Fig. 5). Figure 6 shows the crude and multivariate-adjusted odds ratios for a history of cardiovascular disease according to ABSI. After adjustment of various confounders, an ABSI value higher than the cutoff value remained as an independent predictor of a history of cardiovascular disease regardless of gender.

## Discussion

ABSI was derived empirically from the US NHANES population database as waist circumference divided by a power law approximation of the expected value of waist circumference for weight and height^18^. We found a negligible correlation of ABSI and BMI, indicating that ABSI should be valid as originally defined for application to our subjects. In the present study, we demonstrated that abdominal obesity evaluated by ABSI was negatively correlated with FMD and that ABSI had a better predictive value of endothelial dysfunction than did BMI. We also confirmed that ABSI values above the cutoff values (0.0796 for men and 0.0823 for women), which were derived from ROC curve analysis, were significantly associated with endothelial dysfunction even after adjustment for various confounders. To our knowledge, this study is the first study to show ABSI thresholds that can be used to detect patients with endothelial dysfunction.

In the present study, impaired endothelial function was positively correlated with ABSI in both sexes. It is well known that abdominal obesity and endothelial dysfunction share many common risk factors. Indeed, our results support the results of previous studies showing that higher ABSI is associated with a higher prevalence of cardiovascular risk factors^20,24^. Therefore, we evaluated the associations between ABSI and endothelial function after adjustment of cardiovascular risk factors. We confirmed that ABSI values above the cutoff values were independently associated with endothelial dysfunction in men and women. These findings suggest that high ABSI is an independent risk factor for endothelial dysfunction.

Several investigators have reported an association of ABSI with risk of mortality from cardiovascular disease^22,25^. However, the cutoff value of ABSI associated with a higher risk of cardiovascular events remained unknown. Christakoudi et al. showed that cutoff values of ABSI for all-cause mortality are 0.0833 for men and 0.0762 for women in the European population^23^. In the present study, ROC curve analysis revealed that ABSI of 0.0796 for men and ABSI of 0.0823 for women were cutoff values of endothelial dysfunction. The cutoff values of ABSI for outcomes might be different between the Japanese population and European population. Nevertheless, we found that the cutoff values of ABSI for a history of cardiovascular disease were similar (0.0811 for men and 0.0824 for women, Fig. 5). These findings indicate that the cutoff values of ABSI for higher risk of cardiovascular events are 0.0796 for men and 0.0823 for women in the Japanese population.

The study subjects were enrolled at 22 university hospitals and affiliated clinics in Japan^26^. Height, weight, BMI, and waist circumference of the study participants were similar to nationally representative data in Japan^27^. ABSI was developed to estimate the health of body shape independently of body size (height, weight, and BMI) from a database for the American population^18^. Some researchers suggested that ABSI should be modified by age and race^28,29^. Indeed, Sato et al. reported that ABSI is significantly associated with the incidence of all-cause mortality in Japanese men, while ABSI has a weak association with the incidence of all-cause mortality in Japanese women^30^. In the present study, there was an inverse relationship between ABSI and height, especially in women. This finding suggests that ABSI is not completely appropriate for the Japanese population. However, we confirmed that ABSI was significantly correlated with waist circumference, Framingham risk score, and FMD, which are surrogate markers of cardiovascular events. Therefore, ABSI is a useful tool for assessing cardiovascular risk in the Japanese population.

There is a growing body of evidence that abdominal obesity is better predictor of cardiovascular risk than is BMI^15,16^. Some possible mechanisms by which abdominal obesity impairs endothelial function have been postulated. Abdominal obesity reflects excess subcutaneous and visceral adipose tissue. In obesity, adipose tissue becomes dysfunctional, leading to the promotion of a proinflammatory response^31^. Chronic inflammation has been shown to play a critical role in endothelial dysfunction through a decrease in nitric oxide bioavailability^32–34^. Our study showed that ABSI has a stronger association than BMI with endothelial dysfunction. ABSI had stronger correlations with waist circumference and cardiovascular risk factors. In addition, ROC curve analysis revealed that the AUC value of ABSI to predict a history of cardiovascular disease was superior to that of BMI (Fig. 5). ABSI in conjunction with BMI might be a useful marker for evaluating the risk of cardiovascular events in both men and women^35,36^.

### Study limitations

The present study has some limitations. First, a definitive causal relationship between ABSI and endothelial function could not determine in this study since this study was a cross-sectional design. Second, we had no information on physical activity. Physical activity is associated with both ABSI and endothelial function^37,38^. We cannot rule out the possibility that physical activity influences the association between ABSI and endothelial function. Future studies are needed to confirm the role of physical activity in the relationships between ABSI and endothelial function. Finally, the inverse association between ABSI and height might reflect the generational difference in height, since ABSI was positively correlated with age in this study. Indeed, over the past century, there has been a larger generational difference in height in Asian countries compared to American^39^. Therefore, age-specific cut-offs or some form of modification of ABSI by age will be required in future studies.

In conclusion, high ABSI is independent predictor of endothelial dysfunction. The results of our study suggest that ABSI calculation should be performed for evaluation of the risk of cardiovascular events.

## Methods

### Subjects

A total of 10,247 Japanese adults (7385 subjects from the FMD-J study and 2862 subjects who underwent a health checkup at Hiroshima University Hospital between August 2010 and August 2016) were enrolled in this study^26^. The FMD-J study was a prospective multicenter study conducted at 22 university hospitals and affiliated clinics in Japan to examine the usefulness of FMD in risk stratification for cardiovascular disease in Japanese subjects^26^. The rationale and design of the FMD-J study have been described previously^26^. Subjects with unclear images of the brachial artery interfaces and subjects without information on ABSI were excluded. From this registry, 8823 subjects were recruited. ABSI was calculated by the following equation: waist circumference (m)/[BMI^2/3^ (kg/m^2^) × height^1/2^ (m)]^18^. Hypertension was defined as treatment with oral antihypertensive agents or systolic blood pressure of more than 140 mm Hg or diastolic blood pressure of more than 90 mm Hg measured in a sitting position on at least three different occasions. Diabetes mellitus was defined as treatment with diabetic medication or fasting plasma glucose level ≥ 6.99 mmol/L and hemoglobin A1c level ≥ 48 mmol/mol^40^. Dyslipidemia was defined as treatment with lipid-lowering agents or low-density lipoprotein cholesterol ≥ 3.62 mmol/L or high-density lipoprotein cholesterol < 1.03 mmol/L or triglyceride ≥ 1.69 mmol/L^41^. Smokers were defined as those who were current smokers. Coronary heart disease included angina pectoris, myocardial infarction, and unstable angina. Cerebrovascular disease included ischemic stroke, hemorrhagic stroke, and transient ischemic attack. Cardiovascular disease was defined as coronary heart disease and cerebrovascular disease. Framingham risk score was calculated by points of risk factors: age, total cholesterol level, high-density lipoprotein cholesterol level, systolic blood pressure, diabetes mellitus, and smoking status^42^.

Hiroshima University ethical committee approved the study protocol. The study was executed in accordance with the Good Clinical Practice guidelines. Informed consent for participation in the study was obtained from all subjects. The protocol was registered in the University Hospital Medical Information Network Clinical Trials Registry (UMIN000012952).

### Research procedure

We measured vascular responses to reactive hyperemia in the brachial artery in all subjects^43^. Additional details are available in the online-only “Data Supplement”.

### Measurement of FMD

FMD was measured by using UNEXEF18G (UNEX Co, Nagoya, Japan) as previously described^44^. Additional details are available in the online-only “Data Supplement”.

### Definition of subjects with endothelial dysfunction

The subjects were divided into four quartile groups based on the FMD value. The subjects with a lower quartile of FMD were defined as subjects having endothelial dysfunction. The cutoff values for endothelial dysfunction were as follows: FMD of less than 3.6% in men and FMD of less than 3.1% in women.

### Statistical analysis

Results are presented as means ± SD for continuous variables and as percentages for categorical variables. All reported probability values were 2-sided, and a probability value of < 0.05 was considered statistically significant. Continuous variables were compared by using ANOVA with Tukey’s test for post-hoc comparisons for multiple groups. Relations between ABSI and variables were determined by Pearson’s correlation analysis. Receiver-operator characteristic (ROC) curve analyses were carried out to assess the cutoff values of ABSI and BMI for predicting endothelial dysfunction. The differences in areas under the curve (AUC) were compared to determine the improvement in discrimination between ABSI and BMI using the method of DeLong et al.^45^. As a post hoc exploratory analysis, we fitted an adjusted cubic spline curve, adjusted for age, BMI, and smokers, to explore the shape of the dose-response relationship between ABSI and endothelial dysfunction. We categorized subjects into two groups according to the cutoff value of ABSI for predicting subjects with endothelial dysfunction: a low ABSI group (< 0.0796 in men and < 0.0823 in women) and a high ABSI group (≥ 0.0796 in men and ≥ 0.0823 in women). Cutoff values were derived from receiver-operator characteristic curves. Multivariable logistic regression analysis was performed to identify independent variables associated with endothelial dysfunction. Adjustment variables for multivariable logistic regression models included age (model 1), age, BMI, and smokers (model 2), and age, BMI, presence of hypertension, dyslipidemia, and diabetes, and smokers (model 3). All analyses were conducted using JMP version 14.0 software (SAS Institute, Cary, NC, USA).

## Supplementary Information

Supplementary Information.

## Abbreviations

ABSI: A body shape index
AUC: Areas under the curve
BMI: Body mass index
CI: Confidence interval
FMD: Flow-mediated vasodilation
ROC: Receiver-operator characteristic
