# Supplementary material for: A body shape index is associated with endothelial dysfunction in both men and women
Source: Sci Rep. 2021 Sep 9;11:17873. doi: 10.1038/s41598-021-97325-0 (PMC8429591; doi:10.1038/s41598-021-97325-0)
Supplement: Supplementary file 1 — Supplementary Information. [file 41598_2021_97325_MOESM1_ESM.doc]

**Online Supplement**

**A Body Shape Index is Associated with Endothelial Dysfunction**

**in Both Men and Women**

Short running head: ABSI and endothelial function

Masato Kajikawa, MD, PhD;^a^ Tatsuya Maruhashi, MD, PhD;^b^ Shinji Kishimoto, MD, PhD;^b^ Takayuki Yamaji, MD;^c^ Takahiro Harada, MD;^c^ Yu Hashimoto, MD;^c^ Yiming Han, MS;^b^ Aya Mizobuchi, MS;^b^ Gaku Aoki, MPH;^d^ Kenichi Yoshimura, PhD;^d^ Kazuaki Chayama, MD, PhD;^e^ Chikara Goto, PhD;^f^ Farina Mohamad Yusoff, MD;^b^ Ayumu Nakashima, MD, PhD;^g^ Yukihito Higashi, MD, PhD, FAHA;^a,b^

^a^ Division of Regeneration and Medicine, Medical Center for Translational and Clinical Research, Hiroshima University Hospital, Hiroshima, Japan

^b^ Department of Cardiovascular Regeneration and Medicine, Research Institute for Radiation Biology and Medicine, Hiroshima University, Hiroshima, Japan

^c^ Department of Cardiovascular Medicine, Hiroshima University Graduate School of Biomedical Sciences, Hiroshima, Japan

^d^ Department of Biostatistics, Medical Center for Translational and Clinical Research, Hiroshima University Hospital, Hiroshima, Japan

^e^ Department of Gastroenterology and Metabolism, Institute of Biomedical and Health Sciences, Graduate School of Biomedical and Health Sciences, Hiroshima University Hiroshima, Japan

^f^ Department of Physical Therapy, Hiroshima International University, Hiroshima, Japan

^g^ Department of Stem Cell Biology and Medicine, Hiroshima University Graduate School of Biomedical Sciences, Hiroshima, Japan

Address for correspondence: Yukihito Higashi, MD, PhD, FAHA

Department of Cardiovascular Regeneration and Medicine,

Research Institute for Radiation Biology and Medicine, Hiroshima University

1-2-3 Kasumi, Minami-ku, Hiroshima 734-8551, Japan

Phone: +81-82-257-5831 Fax: +81-82-257-5831

E-mail: [yhigashi@hiroshima-u.ac.jp](mailto:yhigashi@hiroshima-u.ac.jp)

**Methods**

**Study protocol**

Subjects fasted overnight for at least 12 hours and abstained from caffeine, alcohol, smoking, and antioxidant vitamins on the day of the FMD examination. The subjects were kept in the supine position in a quiet, dark, air-conditioned room (constant temperature of 22°C to 25°C) throughout the study. A 23-gauge polyethylene catheter was inserted into the deep antecubital vein to obtain blood samples. FMD was measured after a supine position had been maintained for 30 minutes. The observers were blind to the form of examination.

**Measurement of FMD**

We evaluated the vascular response to reactive hyperemia in the brachial artery for assessment of endothelium-dependent FMD. A high-resolution ultrasonography (UNEXEF18G, UNEX Co, Nagoya, Japan) was used to evaluate FMD. The protocol for measurement of FMD has been described in detail previously.^1^ Briefly, the longitudinal image of the brachial artery was assessed before and after generation of a vascular response to reactive hyperemia by a 5-min period of forearm occlusion to evaluate FMD. FMD was defined as the maximal percentage change in vessel diameter from the baseline value. The correlation coefficient between FMD analyzed at the core laboratory and participant institutions was 0.84 (P<0.001).^2^

**References**

1. Kajikawa, M. *et al.* Combination of Flow-Mediated Vasodilation and Nitroglycerine-Induced Vasodilation Is More Effective for Prediction of Cardiovascular Events. *Hypertension* **67**, 1045-1052, doi:10.1161/hypertensionaha.115.06839 (2016).
2. Tomiyama, H. *et al.* Reliability of measurement of endothelial function across multiple institutions and establishment of reference values in Japanese. *Atherosclerosis* **242**, 433-442, doi:10.1016/j.atherosclerosis.2015.08.001 (2015).

**Supplemental Tables**

Table S1. Clinical Characteristics of Men on the Basis of a Body Shape Index

| Variable | <0.0763  (n=1692) | 0.0763-0.0786  (n=1750) | 0.0787-0.0813  (n=1688) | ≥0.0814  (n=1643) | P value  for Trend |
| --- | --- | --- | --- | --- | --- |
| Age, yr | 45±11 | 49±11 | 52±11 | 60±12 | <0.001 |
| Body mass index, kg/m^2^ | 23.6±3.2 | 23.9±3.1 | 23.9±3.3 | 23.7±3.4 | 0.04 |
| Height, m | 1.70±0.06 | 1.70±0.06 | 1.70±0.06 | 1.68±0.07 | <0.001 |
| Weight, kg | 68.3±10.7 | 68.9±10.2 | 68.8±10.6 | 67.0±11.0 | <0.001 |
| Waist circumference, cm | 79.2±7.7 | 83.6±7.4 | 86.4±7.9 | 90.2±8.9 | <0.001 |
| Systolic blood pressure, mmHg | 126±15 | 128±15 | 130±16 | 130±17 | <0.001 |
| Diastolic blood pressure, mmHg | 78±12 | 81±12 | 81±12 | 79±11 | <0.001 |
| Heart rate, bpm | 63±10 | 64±10 | 65±11 | 66±11 | <0.001 |
| Total cholesterol, mmol/L | 5.12±0.85 | 5.22±0.83 | 5.25±0.93 | 4.99±0.93 | <0.001 |
| Triglycerides, mmol/L | 1.40±1.04 | 1.56±1.19 | 1.61±1.06 | 1.65±1.30 | <0.001 |
| HDL-C, mmol/L | 1.53±0.41 | 1.47±0.36 | 1.45±0.39 | 1.42±0.39 | <0.001 |
| LDL-C, mmol/L | 2.97±0.75 | 3.08±0.75 | 3.10±0.80 | 2.90±0.83 | <0.001 |
| Glucose, mmol/L | 5.44±1.05 | 5.61±0.94 | 5.77±1.28 | 6.11±1.89 | <0.001 |
| Medical history, n (%) |  |  |  |  |  |
| Hypertension | 516 (31) | 752 (41) | 913 (51) | 951 (66) | <0.001 |
| Dyslipidemia | 680 (40) | 942 (52) | 1071 (59) | 922 (64) | <0.001 |
| Diabetes mellitus | 92 (5) | 140 (8) | 219 (12) | 335 (23) | <0.001 |
| Previous cardiovascular disease | 63 (4) | 119 (7) | 165 (9) | 365 (25) | <0.001 |
| Current smoker | 617 (37) | 658 (36) | 634 (35) | 412 (28) | <0.001 |
| Medications, n (%) |  |  |  |  |  |
| Anti-hypertensive therapy | 259 (15) | 417 (23) | 566 (31) | 783 (54) | <0.001 |
| Any lipid modification therapy | 109 (6) | 201 (11) | 281 (16) | 458 (32) | <0.001 |
| Anti-hyperglycemic therapy | 66 (4) | 89 (5) | 166 (9) | 236 (16) | <0.001 |
| Framingham risk score, % | 6.7±5.9 | 8.6±7.0 | 10.2±8.3 | 12.2±9.6 | <0.001 |
| FMD, % | 6.5±3.1 | 6.1±3.0 | 5.6±3.0 | 4.6±3.0 | <0.001 |
| A body shape index | 0.0740±0.0024 | 0.0774±0.0007 | 0.0799±0.0008 | 0.0842±0.0027 | <0.001 |

All results are presented as mean ± SD.

HDL-C indicates high-density lipoprotein cholesterol; LDL-C, low-density lipoprotein cholesterol; FMD, flow-mediated vasodilation.

Table S2. Clinical Characteristics of Women on the Basis of a Body Shape Index

| Variable | <0.0759  (n=510) | 0.0760-0.0796  (n=510) | 0.0797-0.0843  (n=510) | ≥0.0844  (n=520) | P value  for Trend |
| --- | --- | --- | --- | --- | --- |
| Age, yr | 44±14 | 51±14 | 55±13 | 64±13 | <0.001 |
| Body mass index, kg/m^2^ | 22.4±4.0 | 22.4±4.2 | 22.8±3.8 | 22.9±3.3 | 0.03 |
| Height, m | 1.57±0.06 | 1.56±0.06 | 1.56±0.07 | 1.54±0.07 | <0.001 |
| Weight, kg | 54.9±9.7 | 54.7±10.8 | 55.5±9.9 | 54.1±8.7 | 0.12 |
| Waist circumference, cm | 72.1±8.6 | 76.7±9.2 | 80.2±8.9 | 86.6±8.9 | <0.001 |
| Systolic blood pressure, mmHg | 119±18 | 122±19 | 124±19 | 129±19 | <0.001 |
| Diastolic blood pressure, mmHg | 73±12 | 75±12 | 77±11 | 76±12 | <0.001 |
| Heart rate, bpm | 65±10 | 67±12 | 67±11 | 68±11 | <0.001 |
| Total cholesterol, mg/dL | 5.09±0.91 | 5.22±0.91 | 5.28±0.83 | 5.25±0.98 | <0.001 |
| Triglycerides, mg/dL | 0.95±0.60 | 0.99±0.61 | 1.26±0.75 | 1.29±0.68 | <0.001 |
| HDL-C, mg/dL | 1.78±0.44 | 1.76±0.39 | 1.68±0.39 | 1.66±0.41 | <0.001 |
| LDL-C, mg/dL | 2.90±0.78 | 3.05±0.83 | 3.08±0.75 | 3.05±0.88 | 0.006 |
| Glucose, mg/dL | 5.27±1.17 | 5.38±1.05 | 5.44±1.05 | 5.83±1.78 | <0.001 |
| Medical history, n (%) |  |  |  |  |  |
| Hypertension | 162 (30) | 141 (42) | 211 (52) | 531 (70) | <0.001 |
| Dyslipidemia | 168 (31) | 142 (42) | 214 (53) | 525 (69) | <0.001 |
| Diabetes mellitus | 37 (7) | 43 (13) | 43 (11) | 198 (26) | <0.001 |
| Previous cardiovascular disease | 10 (2) | 16 (5) | 23 (6) | 120 (16) | <0.001 |
| Current smoker | 45 (8) | 28 (8) | 35 (9) | 53 (7) | 0.70 |
| Medications, n (%) |  |  |  |  |  |
| Anti-hypertensive therapy | 114 (21) | 95 (28) | 170 (42) | 465 (61) | <0.001 |
| Any lipid modification therapy | 52 (10) | 64 (19) | 87 (21) | 306 (40) | <0.001 |
| Anti-hyperglycemic therapy | 19 (4) | 28 (8) | 26 (6) | 151 (20) | <0.001 |
| Framingham risk score, % | 3.7±4.4 | 4.9±5.0 | 6.0±5.4 | 8.4±6.0 | <0.001 |
| FMD, % | 7.2±3.6 | 6.2±3.8 | 6.0±3.4 | 4.4±3.2 | <0.001 |
| A body shape index | 0.0726±0.0027 | 0.0779±0.0011 | 0.0818±0.0014 | 0.0888±0.0035 | <0.001 |

All results are presented as mean ± SD.

HDL-C indicates high-density lipoprotein cholesterol; LDL-C, low-density lipoprotein cholesterol; FMD, flow-mediated vasodilation.

**Supplemental Figure**

**Figure S1**


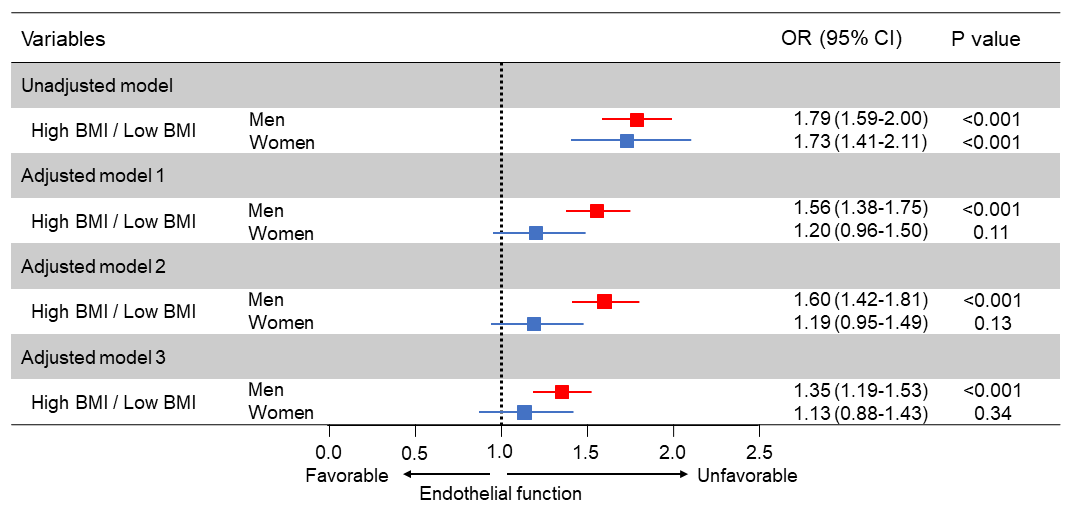


Odds ratios and 95% confidence intervals for a low quartile of flow-mediated vasodilation (FMD) of high body mass index (BMI) group using the low BMI group as the reference. Low quartile of FMD indicates less than 3.6% in men and less than 3.1% in women. Low BMI indicates less than 23.2 in men and less than 22.4 in women.

Model 1: Adjusted for age.

Model 2: Adjusted for age, a body shape index (ABSI), and smokers.

Model 3: Adjusted for age, ABSI, presence of hypertension, dyslipidemia, and diabetes, and smokers.
